# Distant Landmarks Used for Navigation by Homing Pigeons

**DOI:** 10.3390/life16060933

**Published:** 2026-06-01

**Authors:** Martin Wikelski, Dina K. N. Dechmann, Michael Quetting, Georg Heine, Wolfgang Fiedler, Heidi Schmid, Gil Bohrer, Anna Gagliardo

**Affiliations:** 1 Department of Migration, Max Planck Institute of Animal Behavior, 78315 Radolfzell, Germany; ddechmann@ab.mpg.de (D.K.N.D.); mquetting@ab.mpg.de (M.Q.); georg.heine@uni-konstanz.de (G.H.); fiedler@ab.mpg.de (W.F.);; 2 Department of Biology, University of Konstanz, 78464 Konstanz, Germany; 3 Civil Environmental and Geodetic Engineering, The Ohio State University, Columbus, OH 43210, USA; bohrer.17@osu.edu; 4 Department of Biology, University of Pisa, 56126 Pisa, Italy; anna.gagliardo@unipi.it

**Keywords:** navigation, orientation, *Columba livia*, boundary layer, homing flight, stratus fog, mountain range, Alps, olfactory, magnetic, distant landmarks

## Abstract

How animals navigate using single or several sensory modalities remains disputed. Humans often use distant landmarks such as mountain peaks as visual cues for orientation. Whether animals can also rely on distant visual cues while excluding other modalities, such as smell or visual maps, is difficult to test under natural conditions. We investigated this by releasing trained homing pigeons above thick ground fog from a plane. Birds were first trained to home individually from two ground sites, each ~15 km from their loft. Subsequently, trained individuals were released from a plane ~400 m above the terrestrial release sites during sunny conditions. This was followed by two experimental release conditions above continuous ground fog. Above the fog, pigeons cannot use a map or olfactory location cues, but may potentially use magnetic cues. When all landmarks were hidden by ground fog, pigeons showed poor orientation, tortuous flight paths, and descended through the fog within four min, advancing only ~0.1 km (0.1% of the distance) towards home. They subsequently waited for solar cues to continue homing. By contrast, when distant landmarks (alpine mountain tops) were visible, pigeons maintained strong homeward orientation, flying for 20 min and covering 96% of the distance to home, similar to ground-level controls (17 min, 80%). These results demonstrate that when known olfactory cues or local maps are excluded, distant landmarks—but not magnetic cues—enable efficient short-distance homing in experienced pigeons.

## 1. Introduction

Navigation over large spatial scales often relies on the use of distant landmarks that remain visible across wide areas and provide stable directional information. In humans, distant visual features such as mountain ranges, coastlines, skylines, or prominent geological formations play a central role in large-scale orientation and wayfinding, particularly in unfamiliar or sparsely cued environments [1,2,3,4,5,6,7,8]. Because such landmarks change little with observer position, they provide robust allocentric reference points that can anchor cognitive maps and support long-range route planning across kilometers or more [9,10].

The use of visual landmarks is widespread across animal taxa [11,12,13,14,15,16,17,18,19]. Mammals navigating open landscapes, marine animals orienting relative to coastlines, and insects using skyline profiles all exploit large-scale visual features that extend beyond the immediate vicinity, e.g., [20,21,22,23]. In birds, large-scale visual cues have long been suspected to contribute to orientation and homing, particularly in species that repeatedly travel between fixed locations [24]. However, in free-ranging animals, distant landmarks are almost always embedded within a complex sensory environment that also includes local visual cues, olfactory information, wind-related cues, and geomagnetic signals, making it difficult to isolate their specific role.

Here, we use a novel experimental paradigm to disentangle visual, olfactory and magnetic information during small-scale (local homing) navigation: by releasing homing pigeons above ground fog, we abolish ground visual and known olfactory cues. Birds can only use magnetic cues or distant landmarks if they are available. We can thus directly resolve a long-standing debate, i.e., whether pigeons use magnetic cues or distant landmarks in the absence of potential olfactory cues.

Homing pigeons (*Columba livia*) provide a well-established model for studying large-scale navigation [25,26]. For many years, most of the investigations on pigeon navigation focused on the nature of the cues used by birds displaced to unfamiliar sites for determining their position with respect to the goal, so that the sensory basis of the navigational map by homing pigeons became a hot debated issue, mainly between the proponents of the olfactory map versus the proponents of the magnetic map [27,28,29,30]. However, magnetic manipulation of any kind on homing pigeons never affected their homing performance [31]. Conversely, a large body of evidence collected both with traditional (recording both initial orientation at the release site and homing performances) and satellite tracking methods supported a critical role of olfactory cues for pigeon homing from unfamiliar sites [26,31,32,33,34]. While pigeons prevented from smelling local odors both at an unfamiliar release site and in flight after release are impaired at homing [26,33,35], the behavior of anosmic pigeons is comparable to that of control birds if they are familiar with the release site [36,37,38], as familiar visual landmarks provide sufficient navigational information for guiding birds back home.

While the importance of local landmarks of the familiar overflown landscape is well documented [11,13,16], the contribution of distant visual features—such as mountain ridges or large-scale topographic structures visible from afar—remains less clearly understood. One major reason is that distant and local landmarks are typically perceived together, preventing a clear separation of their respective contributions.

Testing the use of distant landmarks in the wild therefore presents a substantial methodological challenge. To assess their role unambiguously, nearby visual and olfactory cues must be removed to eliminate the use of memory while leaving distant features intact. A rare natural solution to this problem is provided by releases above continuous ground fog. During the occurrence of a dense fog layer, local ground-based visual cues are completely obscured. Depending on the weather conditions, elevated distant features, such as mountain ranges, may remain visible above the fog, allowing long-distance visual landmark use. This creates a natural experimental situation in which distant visual landmarks can be selectively present or absent, without altering the geographic position of the release site itself.

Releases above fog also modify the availability of non-visual navigational cues. Ground fog typically marks the upper limit of the atmospheric boundary layer, above which vertical mixing of air is strongly reduced. As a consequence, olfactory cues originating from the landscape below—known to play a major role in pigeon navigation near the ground—are largely unavailable above the fog layer [26,34]. In contrast, geomagnetic cues remain accessible and are not disrupted by altitude differences in this magnitude [39]. Thus, releases above fog provide a unique opportunity to examine visual navigation under conditions in which olfactory information is minimized while magnetic information remains intact.

In the present study, we used this natural separation of sensory cues to test whether homing pigeons can use distant visual landmarks for navigation. Birds were first trained to home from familiar ground-based release sites. They were then transported by aircraft and released above the same sites under three conditions: clear air, fog with distant landmarks visible above the fog, and fog with distant landmarks obscured. By comparing homing performance across these conditions, we directly assessed the contribution of distant visual landmarks to pigeon homing while controlling for release-site familiarity and the availability of non-visual navigational cues.

### Hypotheses and Predictions

We tested the hypothesis that homing pigeons are able to use distant visual landmarks as part of their large-scale navigational system. If distant landmarks contribute to homing, we predicted that pigeons released above fog with visible distant landmarks would show faster homing, more direct routes, or higher homing success than pigeons released above fog when distant landmarks were not visible. Conversely, if distant landmarks are not used, homing performance should not differ between fog conditions, provided that magnetic cues remain available. Additionally, if olfactory cues are essential for navigation at this spatial scale, we predicted an overall reduction in homing performance for all releases above fog compared to ground-based releases, irrespective of distant landmark visibility.

## 2. Materials and Methods

### 2.1. General Setup and Experimental Background

**Figure 1:**
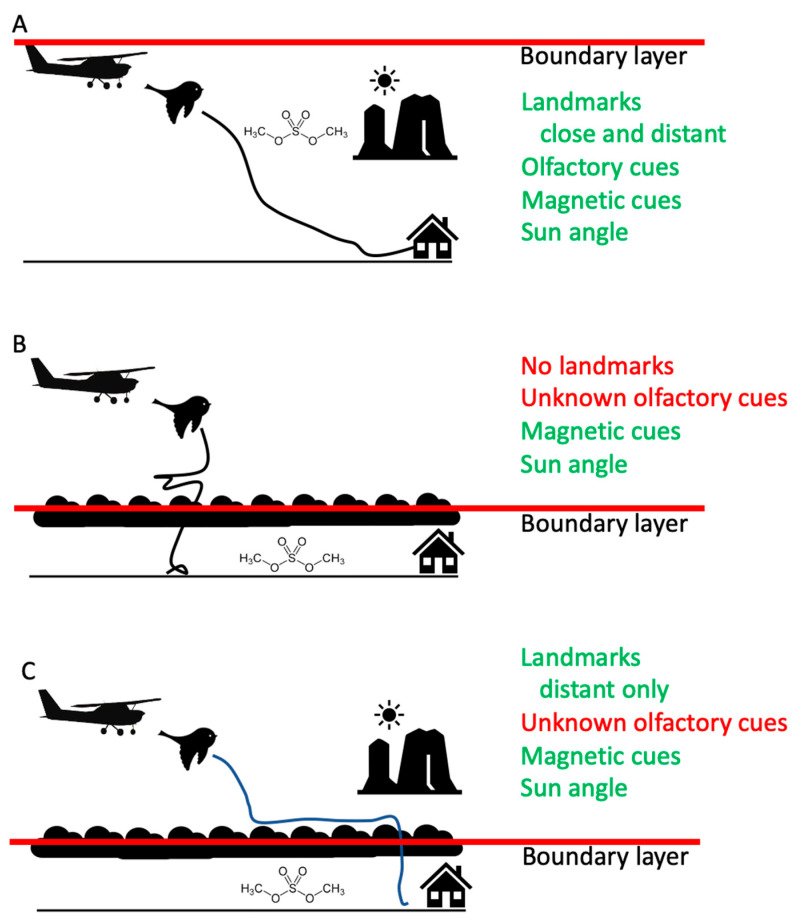
Possible use of environmental cues for homing flights during the experiment. Green text shows available environmental cues, and red text shows unavailable cues. Pigeons were released from a plane. The curved line indicates the average flight direction of the pigeons towards their loft (house icon). Stylized mountains and sun show when these cues were available, respectively. Black cloud symbols indicate thick ground fog, and the horizontal red line highlights the altitude of the atmospheric boundary layer. The chemical formula for dimethylsulfate exemplifies possible chemical, i.e., olfactory, cues available to experienced pigeons at the appropriate atmospheric level below the first boundary layer. (**A**) During regular homing flights, when birds are released from a plane from known locations, pigeons could use a combination of environmental navigational cues. (**B**) When released from a plane above thick ground fog and when no distant landmarks are visible, pigeons can only use magnetic cues or the sun as navigational tools. Because the pigeons are released above the atmospheric boundary layer, the usual olfactory cues are not available to birds. (**C**) Under similar conditions as in (**B**), but when distant landmarks were available, the birds have the sun, magnetic cues and distant landmarks available as navigational cues.

**Figure 2:**
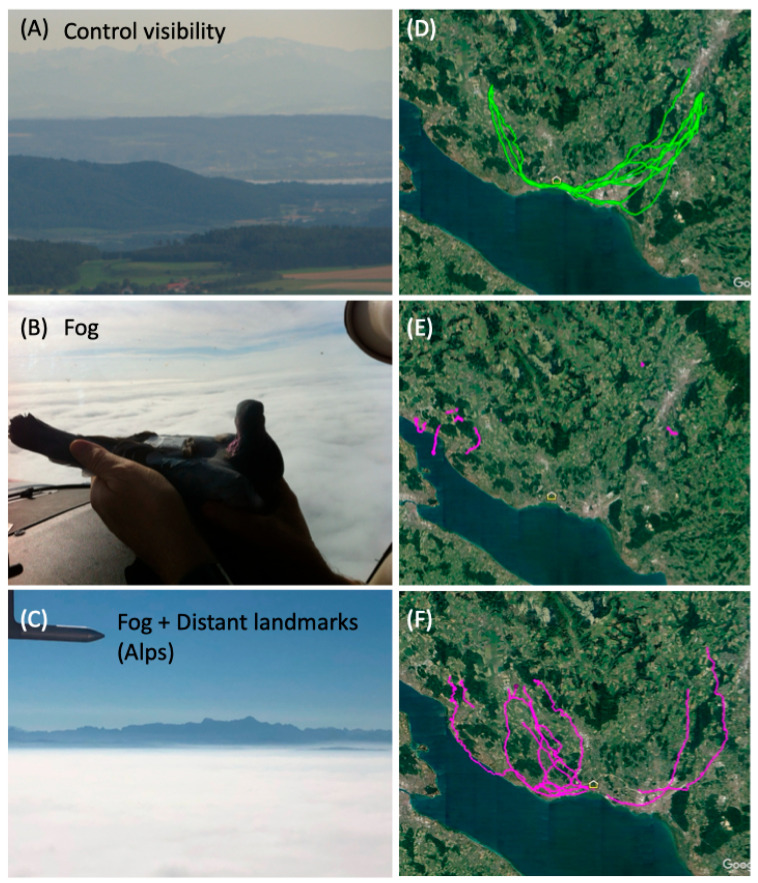
Release conditions and flights of pigeons until the first landing. (**A**) Pigeons were trained to home from two known locations during bright weather conditions with distant landmarks (the Alpine mountain chain) visible. Bright conditions also prevailed during their experimental release from a plane. (**B**) Pigeons (held in hands on top of the Cessna plane cockpit by the experimenter on the co-pilot seat) were released above thick ground fog, but in bright sunlight, without view of distant landmarks. (**C**) During another occasion, the same pigeons were released above thick ground fog, but with clear visibility of distant landmarks. (**D**) Trajectories of pigeons released under control conditions (**A**) from the plane at one of two locations to which they were previously trained. (**E**). Trajectories of pigeons released above thick ground fog without distant landmarks. Note that pigeons do not home and rather descend through the fog to the ground and perch until the ground fog disappears. (**F**) Trajectories of pigeons released above thick ground fog while distant landmarks were visible.

When homing pigeons are trained from a certain location, i.e., when birds home repeatedly from a known point, they orient along specific landmarks such as forest edges or highways (Figure 1A) and become highly efficient in their homing performance. We made use of this situation by allowing birds to home from two terrestrial sites, and then having to home from these sites above a fog layer (Figure 1B) when they did not know (based on ground landmarks) at which site they were located (when released randomly with respect to the release site from the air). After training and experimentally releasing the birds from the ground, we released the pigeons above the atmospheric boundary layer. Under such conditions, there exists a temperature inversion below which intense cloud formation (thick ground fog) occurs (Figure 2B). Above this inversion, which marks the first atmospheric boundary layer, there can be (and always was) bright sunlight and either excellent, or limited visibility (Figure 1B vs. Figure 1C). During excellent visibility conditions, one can see landscape feature up to 200 km distance (Figure 2C), while during limited visibility conditions, distant landscape features are completely occulted (Figure 2B). Under such temperature inversion conditions, one can conduct experiments above the first boundary layer at only several hundred meters above ground, while under non-inversion conditions, the first atmospheric boundary layer is often at least 1500–5000 m above ground (Figure 2A). Interestingly, the air above the first boundary layer is almost never experienced by most birds that fly low to the ground, such as homing pigeons. Thus, the chemical composition of the air above the first boundary layer is not known or familiar to homing pigeons and cannot be used as a potential navigational guidance. However, the sun angle and magnetic information is unaffected by the boundary layer, and if useful for navigation, it could be utilized by the pigeons for navigating home.

### 2.2. Birds and Experimental Procedures

A total of 10 homing pigeons (*Columba livia*), approximately 12–18 months old, were used in this study. All birds were hatched, raised, and housed at a local homing pigeon station near Lake Constance (distance to shore: 300 m) at the village of Fischbach, southern Germany (approx. 47.6705° N, 9.4098° E). Pigeons were maintained as free-flying birds and handled in accordance with German and European regulations governing animal welfare.

The birds were trained to return home individually during sunny, non-windy conditions (wind speed on the ground <5 m/s) by releasing them 10 times on the ground from two different locations during the spring and summer (May to July) of 2014: NW (12 km, 327 degrees) of the loft (approx. 47.7613° N, 9.3213° E), and NE (17 km, 58 degrees) of the loft (approx. 47.7545° N, 9.6112° E). Each bird was then released individually from a Cessna plane flown ca. 400 m over these terrestrial release sites. The time between individual aerial releases was five minutes and that between terrestrial releases was 10 min, to ensure that no grouping of birds during homing flights could occur. The 4-seater Cessna plane (Cessna 172 Skyhawk) took off from Donaueschingen Airfield (EDTD) with two or three people (pilot: M.W., animal handler 1 and 2: various) and 10 pigeons in a transportation crate securely strapped into the passenger seats in the back. Once at the release site, pigeons were removed from the crate and allowed to view the landscape for ca. 5 min (Video S1). At the moment of the release, the plane was shortly stalled to ca. 50 km/h speed and the bird were then held out of the window. All birds flew off immediately and never had any trouble getting away from the aerial influence of the plane (Videos S2, S3, S5 and S6). As the plane needed to constantly move in the air, this resulted in release sites for individual birds that were not exactly above the terrestrial release sites, but within a few hundred meters when projected on the ground. For each bird, the release site GPS location was recorded at the time when the pigeon handler released the bird from their hand, holding it out of the Cessna window.

The altitude of 400 m above ground was chosen based on our previous experience representing an altitude with clear conditions above the ground fog that prevails in this lake basin area in fall and winter (Video S4). Thus, birds were released from the same altitude above the terrestrial release sites during both sunny and foggy conditions (Video S7). Each bird was released once from above each of the two terrestrial release sites during sunny conditions.

Experimental releases from the air took place during the fall period of 2024 when conditions of complete ground fog in the entire Lake Constance basin prevailed for at least from morning to midday. Individual pigeons were randomly assigned to one of the release sites, and the release order during the flights was randomly chosen. Each bird experienced both fog conditions with or without distant landmarks, but in a random order.

To habituate pigeons to carrying equipment, all birds were fitted with a dummy load 20 days prior to their first terrestrial releases. The dummy was comparable in size, shape, and mass to the GPS data logger used during the experiments. It was attached to the birds’ backs using a Velcro^®^ strip glued to trimmed feathers, allowing secure attachment while minimizing discomfort or interference with flight. Birds flew freely with the dummy during the habituation period, ensuring normal flight behavior at the time of testing. The control homing flights (last flight from the ground training as well as both flights during the sunny condition aerial release) were recorded with GPS data loggers, as were both experimental flights during foggy conditions with and without distant landmarks. As expected for well-trained pigeons, during control flights, each bird flew home almost immediately without stopping along the way. Their return time to the loft or the vicinity of the loft, in case the bird was starting to perch after a continuous flight, was recorded.

### 2.3. GPS Data Loggers

We used miniature GPS data loggers (GPS cell phone loggers built in-house or by the University of Konstanz Technical Engineering workshop; GPS cell phone loggers from Fleetronic GSM, Belgium (company not in existence as of 2024), weight 25 g; sampling rate 1 fix every minute) to record the positional data of flying birds with an accuracy of ~4–10 m [30]. The positional fixes stored by the GPS data loggers include latitude, longitude and time of recording. The devices also provide information about altitude, but with insufficient precision to allow a reliable analysis. The tracks for each pigeon for each recorded release were visualized with Movebank and MapInfo (Dalkeith, UK).

#### Quantitative Analyses and Statistical Procedures

Using each pigeon’s GPS position when the bird was perching down for the first time after release, we calculated five homing performance parameters. Flight duration represents the time between an individual’s release, either on the ground or from the plane, and the time when this individual first perched for more than 5 min. Flight distance was again calculated for each individual based on its release GPS site. On the ground, this was one of two terrestrial release sites. From the air, the release sites differed slightly between individuals as the plane had to be in constant motion; thus, we could not release all birds at the exact location. The flight distance was then calculated as the beeline between the release GPS location and the GPS location of the first perching. From these two parameters, we calculated the homing percentage, i.e., the beeline distance to the first perching location divided by the entire distance to the loft, expressed in percent of the entire distance. Finally, the Distance to home was calculated as the absolute beeline distance between the first perching site and the home loft.

We also used an established homing flight parameter named the homing efficiency index (HEI), which takes into account whether a bird approaches or gets away from home during its flight. The HEI is defined as:

$$HEI=\frac{a}{l}\times \frac{\left(b-c\right)}{b}$$

where a is the beeline between the release site and the last fix recorded, l is the track length (as in [28,29]), b is the beeline between the release site and home, and c is the beeline between the last fix recorded and home (all in km). Consequently, if the bird failed to approach home, then HEI < 0; if the bird approached home, then HEI > 0; and if the bird homed, HEI = 1. The various values of the three groups were compared with an ANOVA test, and the Dunn’s test was used for multiple comparisons. We used SPSS 31.0 for statistical analysis.

## 3. Results

### 3.1. General Observations

After 10 training flights from two locations located 12 NW or 17 km NE away from the loft, all 10 birds individually showed high homing performance during an experimental terrestrial release (Figure 2A,D). This is expected for trained homing pigeons.

When the same birds were released above thick ground fog (Figure 2B, Videos S4 and S6), they did not attempt to home but rather descended through the fog layer and landed on the ground. As there was thick fog covering all altitudes down to the ground, the birds did not attempt to fly below the fog (which was not possible), but rather perched almost immediately when they were close to the ground (Figure 2E). All of these birds later returned home to the loft, but only when the sun reappeared and the fog had retracted or disappeared.

In contrast, when distant visual landmarks (the Alpine chain of mountain tops) were visible upon release from the plane (Figure 2C, Video S3), the birds first descended down to several meters to tens of meters above the fog stratus layer (Video S7). From there, they continued their homing flights along similar trajectories compared to their previous control flights from the ground (Figure 1). Although their homing was not perfect, most of the pigeons ended up very close to their home loft before perching for the first time after a continuous flight.

### 3.2. Quantifying the Homing Efficiency Index (HEI)

**Figure 3:**
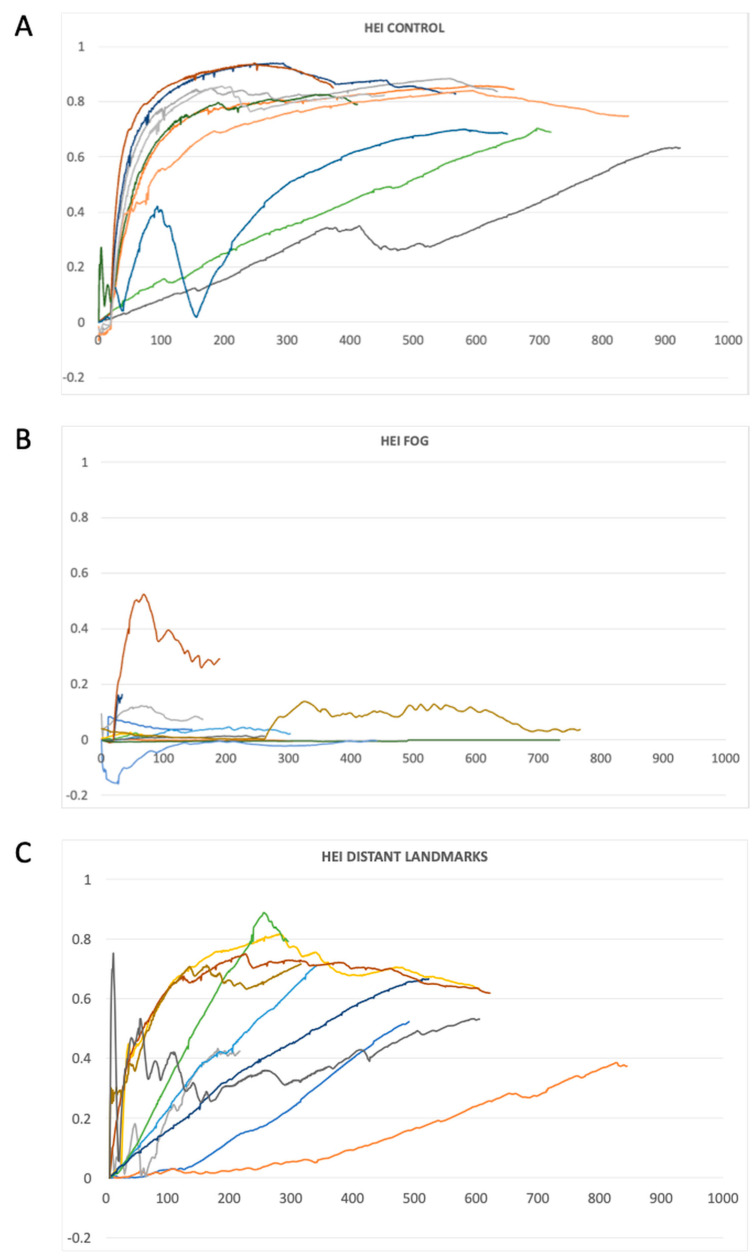
Homing performance of pigeons released from known locations from a plane. (**A**) Homing efficiency index (HEI) of pigeons released during bright control conditions. (**B**) HEI of pigeons released above thick ground fog in bright sunlight, but with no visibility of distant landmarks. (**C**) HEI of pigeons released above thick ground fog in bright sunlight, with good visibility of distant landmarks. Each colored line indicates the flight of one individual pigeon.

**Figure 4:**
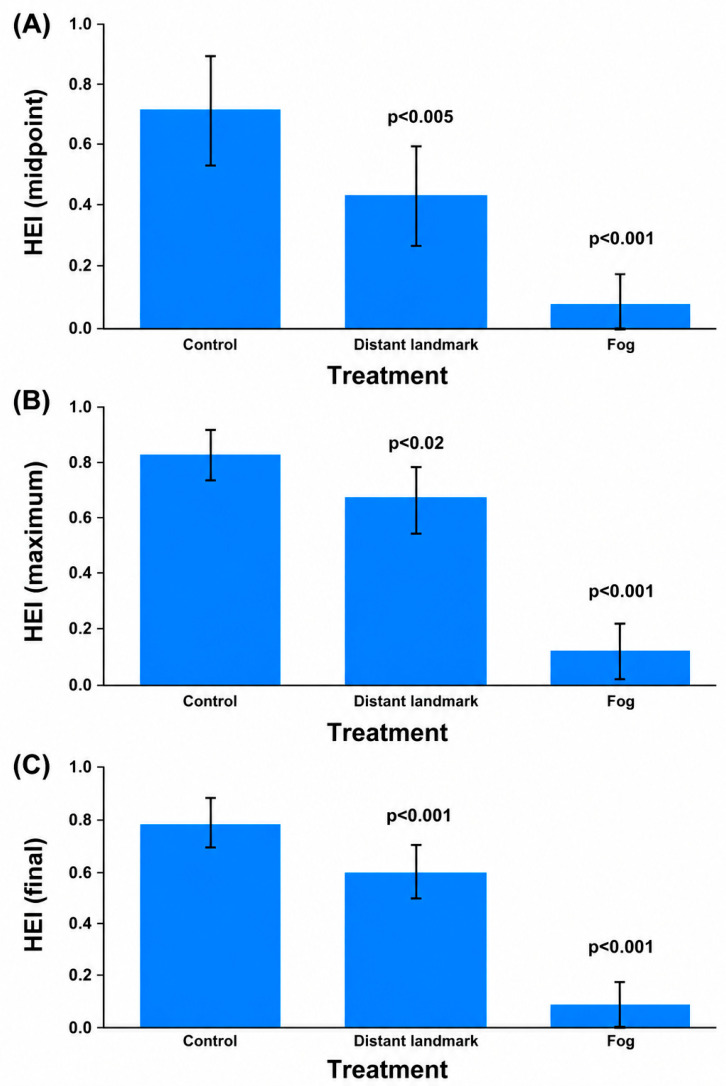
Homing performance of pigeons released from known locations from a plane, separated into the control group, the group seeing distant landmarks, and the group only exposed to thick ground fog. Data show group means ±95% confidence intervals and *p*-values based on LSD tests after ANOVA comparisons. (**A**) Homing efficiency index (HEI) of pigeons during the midpoint of individual tracks. (**B**) Maximum HEI of individual pigeons. (**C**) The final HEI of pigeons. Each colored line indicates the flight of one individual pigeon.

The HEI has been introduced to quantify the homing performance of pigeons and other animals, and was previously employed successfully to characterize such whole-animal performance parameters in natural settings [30]. When our pigeons were released on the ground after 10 training flights from each site, the showed—on average—a very strong and sharply increasing HEI over time (Figure 3A). Not all individuals were equally performant as expressed by their HEI; some individuals, in fact, showed low HEIs for a long time and only appeared to home at a time when the first individuals were already near home or had already reached the loft. Such a variety is, however, expected based on individual performance differences seen in all previous GPS tracking studies on homing in pigeons (for a quantitative comparison between experimental treatments, see Figure 4). We use analysis of variance to test whether the HEI differs between different experimental settings. Because we randomized the sequence of treatments, we do not include this in the analysis as a covariate, nor do we include potential individual behavioral differences based on body weight, age, or sequence during a release event.

When the individuals were released from the plane above thick ground fog without visible landmarks (Figure 3B), all except one bird showed HEIs close to zero, implying that the birds did not attempt to home and made no spatial advances towards home during the time they were observed (i.e., here until they first perched and thus their GPS position remained stationary within a radius of 20 m).

In contrast, when birds were released from the plane above thick ground fog but with visibility of distant landmarks, they displayed high final HEIs (until they perched for the first time) and steep increases in HEI over time. Again, we observed the expected variability in HEI based on individual homing performance seen in all pigeon homing experiments.

We conclude that trained pigeons can home very efficiently when released 400 m above their release location from a plane.

### 3.3. Statistical Comparison Between Experimental Situations

To quantify the flight and homing performance of pigeons when they were subjected to the experimental situations described above, we performed ANOVA analyses with LSD post hoc comparisons between groups.

The homing efficiency index (HEI) of pigeons during the midpoint of an individual return flight differed between treatments, with the highest HEIs under control conditions and the lowest HEIs—indistinguishable from chance directions—under thick ground fog conditions (Figure 4A, ANOVA, F = 25.8, df = 30, *p* < 0.001, LSD as indicated). Similarly, maximum HEIs and final HEIs differed between treatments (Figure 4B, ANOVA, F = 66.1, df = 30, *p* < 0.001, LSD as indicated, and Figure 4C, respectively, ANOVA, F = 123.9, df = 30, *p* < 0.001, LSD as indicated).

**Figure 5:**
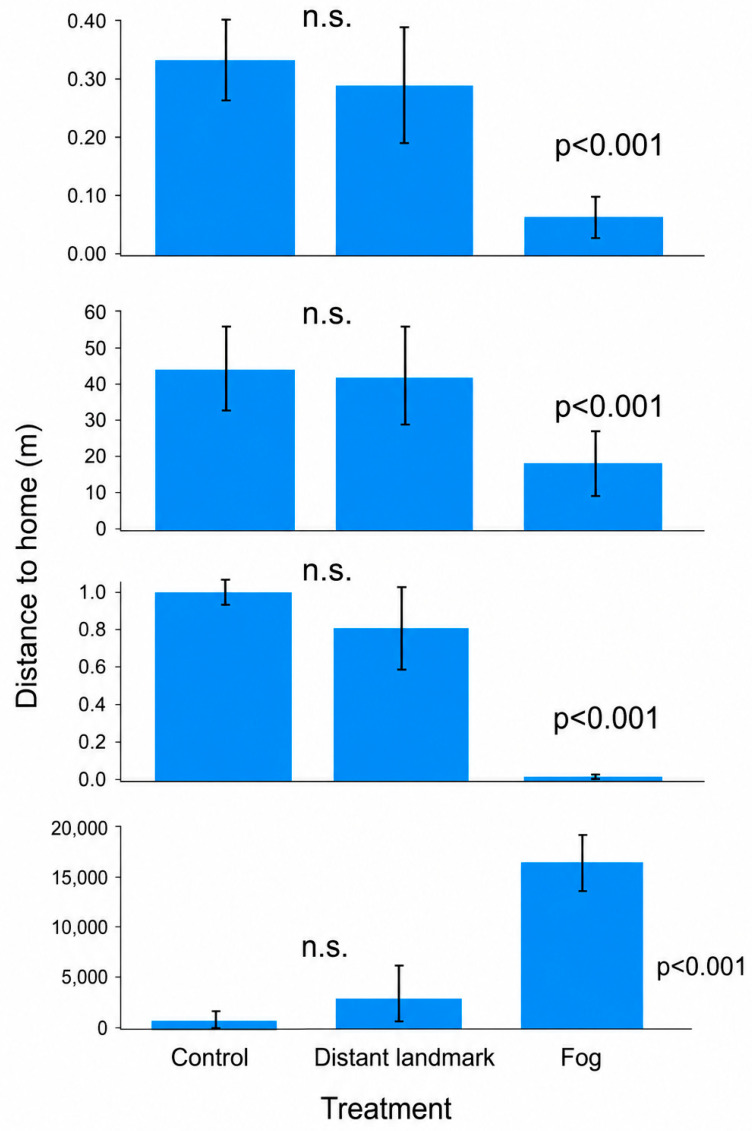
Homing performance of pigeons released from known locations from a plane. Data show group means ±95% confidence intervals and *p*-values based on LSD tests after ANOVA comparisons. Only during complete foggy overcast conditions without visual landmarks, pigeons showed short flight durations, short flight distances, low homing percentages and perched after continuous flight far away from their home loft. The performances of control birds and pigeons released from a plane above thick ground fog with distant landmarks visible to them did not show any statistical differences in any of the above parameters.

The flight duration of individuals was similar between control and fog-with-landmark conditions, but dramatically declined during fog-no-landmark conditions (ANOVA, F = 19.6, df = 30, *p* < 0.001, LSD significant as indicated in Figure 5, top panel).

Pigeons did not fly long distances when they could not see distant visual landmarks, and rather descended through the ground fog to immediately perch when getting close to the ground layer (2nd panel from top, ANOVA, F = 36.5, df = 30, *p* < 0.001, LSD significant as indicated in Figure 5).

The homing performance of pigeons, expressed as the percent of distance an individual covered to approach home, was significantly lower in pigeons when they did not see distant landmarks while being released above thick ground fog (Figure 5, 3rd panel). In contrast, pigeons that were exposed to distant visual landmarks were able to show high homing percentages (ANOVA, F = 114.3, df = 30, *p* < 0.001, LSD significant as indicated in Figure 3).

Finally, as the ultimate performance parameter for a homing pigeon, i.e., how far from the home loft the pigeon perched without advancing further during the foggy conditions, it was clear that again the birds exposed to thick ground fog without visible landmarks in the distance did not home well. In contrast, the birds released during sunny control days (ANOVA, F = 85.9, df = 30, *p* < 0.001, LSD significant as indicated in Figure 5, lowest panel).

## 4. Discussion

### 4.1. Use of Distant Landmarks Above the Fog

The central finding of this study is that homing pigeons are able to orient homeward and reach the home area after aerial release above a continuous fog layer, but only when distant visual landmarks are visible [38]. When distant landmarks were obscured, pigeons failed to initiate directed homing and instead descended rapidly through the fog layer to the ground. This striking contrast demonstrates that, under these conditions, distant landmarks are not merely auxiliary cues but appear to be essential for establishing a usable navigational solution [15,40,41,42,43,44,45].

When distant landmarks such as mountain ranges were visible above the fog, pigeons flew home with a precision comparable to control releases under normal ground-based conditions [46,47]. Flight paths were directed, homing success was high, and birds approached the loft area with remarkable spatial accuracy. This indicates that pigeons can rely on far-distance visual features alone to support large-scale navigation, even in the absence of local landmarks and other ground-based cues. The results thus provide direct experimental evidence that distant landmarks can function as primary navigational references rather than as supplementary cues used only near the goal (c.f.) [18,48].

Our results are at first in apparent contrast to Wagner’s [46] experiments, in which homing pigeons were released above opaque stratus over the Swiss Plateau to determine whether they could locate their loft beneath it. Wagner worked under thermic inversion conditions where the birds had ground visibility below the low stratus layer. Birds above the clouds appeared lost, while those that descended beneath them returned home directly. Wagner inferred, via atmospheric propagation modeling, that infrasonic waves virtually transmitted the location of the loft area. Supposedly, these signals would have been ducted beneath the inversion layer and would not have reached the release sites above it. The absence of homeward infrasonic cues above temperature inversions was used to explain the disorientation of Wagner’s birds, especially if such signals are the predominant cues used by pigeons to home. Because Wagner’s birds had ground visibility below the low stratus, it is expected that they home as soon as they descend through the stratus, exactly as Wagner observed.

### 4.2. Absence of Olfactory Information Above the Boundary Layer

A key aspect of the experimental design is that releases above fog occurred above the atmospheric boundary layer [49,50,51,52]. Under such conditions, vertical mixing of air masses is strongly reduced, and landscape-derived olfactory cues are effectively unavailable [26,34]. The complete loss of oriented homing when birds were released above fog without visible landmarks therefore strongly suggests that olfactory navigation cannot operate under these conditions, and birds have to rely upon visual landmarks much more strongly [38].

This finding reinforces earlier work showing that olfaction is critical for pigeon navigation near the ground but also demonstrates its limitations at higher altitudes. The present results indicate that olfactory cues are not only unnecessary when distant familiar landmarks are available, but also insufficient to support navigation when birds are isolated from the ground-level odor mosaic, as predicted [29,33,34,53,54,55,56]. Thus, the data provide a rare field-based exclusion of olfactory navigation, achieved without artificial manipulation of the birds themselves [28,29].

### 4.3. Implications for Magnetic Navigation and the “Map” Problem

The failure of pigeons to orient when released above fog without landmarks also has important implications for magnetic navigation [27,57,58,59], which was, e.g., postulated for sea turtles [60], amphibians [61], salmon [62], European eels [63] and pigeons [64,65]. Magnetic information—both directional and positional—remains available at altitude and is unaffected by fog. Birds released under landmark-free fog conditions were therefore still able to perceive magnetic field parameters and to maintain a magnetic heading. Nevertheless, they did not initiate consistent homing trajectories.

This strongly suggests that magnetic cues alone do not provide pigeons with sufficient positional (“map”) information under these conditions, c.f. [66]. If pigeons possessed a functional magnetic map based on spatial gradients or intersections of magnetic parameters (e.g., total intensity, inclination, declination), they should have been able to determine their approximate location and select an appropriate homeward direction, c.f. [39,60]. The observed behavior instead indicates that, while pigeons may maintain a magnetic compass, they do not know where they are on such a map in the absence of visual or olfactory information. As pigeons experiencing visual landmarks returned home under similar conditions, it is unlikely that any kind of motivational aspect could be involved in their decision to immediately descend into the fog and stay stationary [67].

This distinction between a compass and a map is critical. The data suggest that pigeons released above fog without landmarks possess orientation mechanisms similar to birds in complete darkness and clouds [68] but lack positional knowledge. As a consequence, they appear unable to translate directional information into a navigational decision and instead abandon flight at altitude, descending through the fog to regain access to ground-based cues.

#### Descent Behavior and Decision-Making Under Uncertainty

The rapid descent through the fog layer observed in landmark-free conditions provides insight into how pigeons respond to navigational uncertainty [69,70]. Rather than maintaining altitude and flying in an arbitrary or magnetically defined direction, birds quickly descend to the ground, presumably to re-enter a sensory environment where reliable cues become available. This behavior supports the idea that pigeons actively evaluate cue reliability and switch strategies when navigation becomes unreliable [71].

In contrast, when distant landmarks were visible, pigeons maintained flight just tens of meters above the fog layer. This altitude appears to represent a compromise between remaining close to the visual horizon and avoiding descent into the fog itself. From this position, distant features such as the Alps were clearly visible on days when fog was restricted to the lowlands, whereas on days when fog extended into higher elevations, no such features were available. This natural variability proved crucial for separating the effects of distant landmark visibility, and is in contrast to the conditions experienced by birds in Wagner’s experiments with low stratus layers [46].

### 4.4. Precision of Navigation Using Far-Distance Visual Projections

One of the most striking aspects of the results is the spatial precision achieved by pigeons navigating solely with distant landmarks, known from technical systems [72,73,74]. Even when flying above fog-covered lowlands and across large bodies of water, birds arrived close to their loft. Notably, flight trajectories projected onto the ground showed that pigeons flew over open water (Lake Constance)—something they never do under ground-based conditions with full visibility. Under normal conditions, pigeons avoid the lake, presumably because they can see it and choose to follow the shoreline from further inland.

Above the fog, however, pigeons appear to follow memorized large-scale landmark projections (c.f.) [14] rather than local terrain features. They roughly paralleled the shoreline as learned during training, yet crossed over the lake because the lake itself was visually absent. This indicates that pigeons store and use an abstract representation of landmark geometry that does not depend on continuous visual confirmation of the underlying terrain [13,16,18,75,76].

The precision of this process is remarkable and raises important questions about the spatial resolution of distant landmark representations [77]. How accurately can pigeons encode angular relationships between far-distance features? How stable are these representations across changing viewing angles and altitudes? The present study demonstrates that such precision is sufficient for successful homing, but future experiments will be needed to quantify its limits.

### 4.5. Ethical Considerations and Animal Welfare

An important aspect of this study is that all aerial releases were conducted under conditions that posed no apparent risk to the birds [78]. Releases were performed from a small Cessna aircraft flown at approximately 40 knots (stall speed), a speed well within the comfortable flight range of pigeons [79,80]. Birds exited the aircraft smoothly and showed no signs of distress or disorientation attributable to the release procedure itself.

During training under clear conditions, pigeons repeatedly exhibited anticipatory behavior prior to release, positioning themselves at the aircraft window and visually tracking the landscape below. This behavior strongly suggests that pigeons were actively perceiving and processing visual information during flight and that aerial releases were not aversive. Although the release altitude (approximately 400 m above ground level) exceeded typical cruising heights during routine homing [81], pigeons routinely descended to just above the fog layer, indicating active control over flight altitude and comfort.

### 4.6. Limitations of the Study

A limitation of the present study is the relatively small sample size and the restriction to experienced homing pigeons trained from only two familiar release directions. Although the experimental contrast between fog conditions with and without distant landmarks produced a strong and consistent behavioral effect, the study design does not allow detailed assessment of inter-individual variation, learning history, or the potential contribution of additional cues under more heterogeneous environmental conditions. Furthermore, the atmospheric structure above the fog layer was inferred from established boundary-layer principles rather than measured directly at the release altitude, and the GPS sampling interval limited fine-scale reconstruction of rapid flight maneuvers and altitude changes during descent and orientation.

### 4.7. Avenues for Future Research

Future research should investigate how pigeons encode and recognize distant landmark configurations across different spatial scales, viewing angles, and altitudes. Experiments combining high-resolution GPS and inertial sensors with controlled manipulations of skyline visibility, atmospheric conditions, and magnetic fields could clarify how distant visual cues interact with compass systems and learned route memories. Comparative studies across species, including migratory birds and other long-distance navigators, may reveal whether the use of far-distance visual projections represents a general navigational principle. In addition, integrating behavioral experiments with computational models of panoramic landmark processing could help quantify the spatial precision and stability of landmark-based cognitive maps in freely moving animals.

### 4.8. Broader Implications for Navigation Research

These findings add to a long-standing literature showing that animal navigation is not based on a single dominant sensory mechanism, but on flexible integration of multiple cue systems. In homing pigeons, previous work has emphasized the importance of olfactory cues for map-based navigation from unfamiliar locations, while visual landmarks are known to guide movement across familiar terrain. The present results refine this view by showing that distant visual landmarks can support efficient homing even when local visual information and ground-level olfactory cues are unavailable. Thus, distant landmarks appear capable of functioning not merely as route-following aids near the goal, but as large-scale spatial references that allow pigeons to determine and maintain a homeward trajectory under otherwise cue-deprived conditions.

The results also speak directly to the debate over magnetic map use in birds. Because magnetic cues remained available above the fog layer, the failure of pigeons to orient when distant landmarks were obscured suggests that magnetic information alone was insufficient to generate a usable positional solution in this context. This does not exclude a role for magnetic compass orientation, but it indicates that, at least in experienced homing pigeons navigating over familiar terrain, magnetic cues may not substitute for visual or olfactory map information. The findings therefore support a hierarchical or context-dependent model of navigation, in which animals weigh available cues according to reliability, familiarity, and spatial scale.

More broadly, the study has implications beyond pigeons. Many animals navigate through environments in which local cues are intermittently unavailable, ambiguous, or obscured. Migratory birds may use coastlines, mountain ranges, celestial cues, wind patterns, and olfactory gradients in combination; insects can rely on skyline panoramas and celestial compass cues; marine animals may integrate magnetic, olfactory, acoustic, and visual information depending on habitat. The pigeon results suggest that distant, stable landscape features may be more important than previously appreciated as spatial anchors, especially in species that repeatedly move through large but familiar areas.

These findings also encourage a shift from asking which single cue animals use to asking how animals dynamically combine cue systems under changing environmental conditions. The rapid descent of pigeons when distant landmarks were unavailable suggests active assessment of navigational uncertainty: rather than flying randomly, birds appeared to abandon directed flight until more reliable cues became accessible. Similar decision rules may operate in other taxa, for example, when migrating birds delay departure under overcast skies, insects alter routes when skyline cues are disrupted, or marine animals change movement paths when odor or magnetic information becomes unreliable.

Finally, the study highlights the value of natural experiments for animal navigation research. Fog created a rare situation in which local visual and olfactory cues were removed while distant landmarks were selectively present or absent. Comparable approaches in other systems—using weather, topography, sensory occlusion, or naturally varying cue availability—could help clarify how animals prioritize landmarks, olfaction, magnetism, celestial information, and memory during real-world movement.

Beyond pigeon navigation, these results illustrate how complex navigational traits can be dissected under natural conditions [82,83]. Laboratory experiments, while essential for isolating sensory mechanisms, cannot replicate the full suite of sensory, motor, and environmental interactions involved in real navigation [84]. Navigation necessarily involves movement through space, active sensing, and continuous integration of multiple cues—processes that are difficult to reproduce in stationary or simplified settings [85].

The present study demonstrates that carefully chosen natural conditions, such as fog layers and aerial releases, can provide powerful experimental leverage in the wild. By selectively removing entire classes of cues, it becomes possible to test core assumptions about navigation systems that would otherwise remain inaccessible.

### 4.9. Distant Landmarks as a General Navigational or Orientational Guide

Finally, the findings support a broader view of navigation in which distant visual cues play a central role [86,87]. In addition to fixed landscape features such as mountain ranges, other distant but predictable cues—including the sun, moon, and the Milky Way—may function as large-scale visual references as compass reference, but most likely not as a map [88,89,90]. Birds possess time-compensated sun and moon compasses and are known to orient relative to stellar patterns at night, suggesting that they integrate multiple distant cues into a coherent navigational framework [91].

This parallels human navigation, where distant and often moving celestial cues have historically been used alongside terrestrial landmarks [92]. The present results support the suggestion that pigeons, and likely other birds and homing animals [93], rely on a hierarchy of distant references that together enable large-scale navigation when local cues are unavailable.

## 5. Conclusions

This study shows that homing pigeons can navigate with high precision when released above fog, but only when distant visual landmarks are visible. The results provide evidence against magnetic map-based positioning under these conditions while highlighting the critical role of distant landmarks in establishing positional knowledge. By exploiting natural atmospheric conditions, this work offers a powerful example of how complex navigational systems can be experimentally dissected in the wild and opens new avenues for testing the limits and mechanisms of large-scale animal navigation.

## Data Availability

All original tracking data are available in the Movebank data archive under DOI (will be made available upon acceptance of the manuscript).

## Supplementary Materials

The following supporting information can be downloaded at: https://www.mdpi.com/article/10.3390/life16060933/s1, Video S1: Pigeon observing the landscape under sunny conditions. Video S2: Pigeon release in sunny conditions. Video S3: Pigeon release above fog with landmarks visible. Video S4: Fog, no visual landmarks available for the pigeons. Video S5: Pigeon release above fog without landmarks. Video S6: Fog, no visual landmarks available for the pigeons. Video S7: View from above the fog as a pigeon would see.

## Abbreviations

The following abbreviation is used in this manuscript:

GPS	Global positioning system
